# Factors influencing psychological distress among breast cancer survivors using machine learning techniques

**DOI:** 10.1038/s41598-024-65132-y

**Published:** 2024-07-01

**Authors:** Jin-Hee Park, Misun Chun, Sun Hyoung Bae, Jeonghee Woo, Eunae Chon, Hee Jun Kim

**Affiliations:** 1 College of Nursing, Research Institute of Nursing Science, Ajou University, Suwon, Republic of Korea; 2 Department of Radiation Oncology, School of Medicine, Ajou University, Suwon, Republic of Korea; 3 Management Team, Cancer Center, Gyeonggi Regional Cancer Center, Suwon, Republic of Korea; 4 College of Nursing, Ajou University, 164, World Cup-ro, Yeongtong-gu, Suwon, 16499 Republic of Korea

**Keywords:** Breast cancer, Distress, Machine learning, Quality of life, Distress thermometer, Breast cancer, Quality of life, Risk factors

## Abstract

Breast cancer is the most commonly diagnosed cancer among women worldwide. Breast cancer patients experience significant distress relating to their diagnosis and treatment. Managing this distress is critical for improving the lifespan and quality of life of breast cancer survivors. This study aimed to assess the level of distress in breast cancer survivors and analyze the variables that significantly affect distress using machine learning techniques. A survey was conducted with 641 adult breast cancer patients using the National Comprehensive Cancer Network Distress Thermometer tool. Participants identified various factors that caused distress. Five machine learning models were used to predict the classification of patients into mild and severe distress groups. The survey results indicated that 57.7% of the participants experienced severe distress. The top-three best-performing models indicated that depression, dealing with a partner, housing, work/school, and fatigue are the primary indicators. Among the emotional problems, depression, fear, worry, loss of interest in regular activities, and nervousness were determined as significant predictive factors. Therefore, machine learning models can be effectively applied to determine various factors influencing distress in breast cancer patients who have completed primary treatment, thereby identifying breast cancer patients who are vulnerable to distress in clinical settings.

## Introduction

Breast cancer is the most common cancer among women worldwide, and South Korea is one of the Asian countries with the highest incidence of breast cancer^1^. The five-year survival rate for breast cancer in South Korea is currently 93.6%^2^. Unlike Europe and the United States, where breast cancer occurrence rates are high among women in their 50 s and 60 s, South Korea has a high proportion of women in their 40 s developing breast cancer. Therefore, helping breast cancer survivors to manage the breast cancer-related health problems that occur after primary treatment and enjoy a high quality of life is critical^1,2^.

Breast cancer patients experience distress as a result of various physical, psychological, and social problems that may arise during treatment^3^. Distress refers to an unpleasant experience that may be physical, mental, social, or spiritual in nature, which may hinder the ability of cancer patients to cope with treatment effectively^4^. The stress experienced by breast cancer patients varies in severity and incidence depending on the time of measurement^5^. However, it is the highest at the time when the cancer is diagnosed, and more than 30% of breast cancer patients experience severe stress even once treatment has been terminated or completed^6,7^. Temporary distress experienced by patients is a normal response; however, prolonged distress degrades their compliance and satisfaction with treatment^8,9^. Distress is also known to interfere with health-related decision making^10^ and decrease physical function, well-being, and quality of life^5,11^, as well as resulting in negative effects throughout the course of cancer treatment^4,9^. Furthermore, distress among cancer patients, which is the sixth vital sign in cancer care, is a key predictor of cancer mortality and quality of life^6^. Therefore, its importance must be recognized in all processes of cancer diagnosis and treatment and it must be monitored, recorded, and managed continuously^7,12^.

To facilitate the successful transition from patient to survivor, the level of distress experienced by patients with breast cancer should be assessed at the initial point of transition from patient to survivor post treatment and the factors influencing it should be identified. This will enable healthcare providers to predict the occurrence of severe distress and provide psychological and social interventions to reduce distress^7^. However, previous studies on distress in breast cancer patients exhibited several limitations, such as primarily focusing only on those who had survived several months to several years after treatment^4,5^ or having limited sample sizes^6^.

In recent years, the integration of artificial intelligence into medical technologies has enabled the analysis and prediction of disease risk factors, as well as research on disease diagnosis and mortality^13,14^. The use of artificial intelligence in the field of healthcare ensures highly accurate and reliable disease diagnosis and prognosis prediction. Machine learning algorithms are particularly useful for effectively extracting and analyzing large volumes of data in exploratory research. Furthermore, machine learning offers the advantage of being relatively unconstrained by the limitations imposed by various assumptions in traditional research methodologies^15^. By simultaneously including several variables, machine learning can determine the relationship among key variables and assess the importance of predictive factors^16^. This study aimed to assess the level of distress in breast cancer survivors and analyze the variables that significantly affect distress using machine learning techniques.

## Methods

### Study design

This cross-sectional survey study aimed to assess the level of distress and determine the factors influencing distress in breast cancer patients who have completed primary treatment for breast cancer.

### Study participants and data collection

A total of 641 adult breast cancer patients aged 19 and above, who were registered at the Cancer Survivor Integration Support Center from April 2020 to July 2022 and satisfied the selection criteria, participated in this study. The selection criteria for participants included women aged 19 to 64 with breast cancer, without any psychiatric issues such as depression or any history of recurrence or metastasis. After completing primary treatment, the breast cancer patients could voluntarily register at the Cancer Survivor Integration Support Center to participate in the cancer survivorship program. A survey was conducted on the level of distress and related factors for patients who agreed in writing to participate after a nurse at the center explained the purpose of the survey. An analysis was conducted using these data collected from the Cancer Survivor Integration Support Center, with specific data selected that met the criteria for participant selection. All personal information, including patient names and hospital identifications, was removed before being provided for analysis. This study was conducted after obtaining approval from the Institutional Review Board of the hospital with which the cancer center was affiliated prior to receiving the data.

### Measures

The National Comprehensive Cancer Network (NCCN) Distress Thermometer is a widely used screening tool for assessing psychosocial distress in cancer patients. When using this tool, patients are asked to circle the number that best describes the amount of distress that they have experienced over the past week and to indicate whether any of the items on the specified problem list have caused problems. The thermometer itself is unspecific; however, the problem list identifies the multidimensional categories that cause distress. The Distress Thermometer consists of an 11-point visual analog scale ranging from 0 (no distress) to 10 (extreme distress) and a 39-item problem list^17^. The patients rate the level of distress that they have experienced over the past week using the visual analog scale. The established cutoff score for further screening is four^12,18^. Patients are then asked to fill in the problem list that accompanies the visual image of the distress thermometer to verify whether (yes/no) they have experienced any of the listed problems over the previous seven days to identify the factors related to the distress^10,17^. The NCCN recommends incorporating the problem list for patients as part of the assessment to assist the provider in identifying sources of patient distress. The problem list consists of 36 problems under the following five grouped categories: spiritual/religious, practical, family, emotional problems, and physical problems^12^.

### Statistical analysis

The χ2 test (Fisher's exact test) and t-test were conducted using the Jamovi program (version 2.3.21) to evaluate the level of distress and survival rate of breast cancer patients according to the distress problem list and the difference between mild and severe distress groups. Training and testing of the machine learning models were performed and the feature importance was verified and visualized using Python version 3.9.16. The specific analysis steps are described in the following.

#### Data preprocessing

Data preprocessing involves organizing data before analyzing them and using them to train models^19^. This process includes handling missing values in the collected data and transforming them into the necessary format for data learning through encoding. In this study, no significant results were observed in the demographic and treatment-related characteristics; therefore, the items in the list of distress problems were processed as dummy variables. Thus, data preprocessing was performed using the Pandas and NumPy libraries in the Python package.

#### Model training

Five machine learning models that are representative supervised learning algorithms for binary classification—Logistic Regression, XGBoost, Random Forest, Support Vector Machine, and CatBoost—were applied. They were used to identify the relationships between categories in existing data and autonomously determine the category of newly observed data^19^. This study aimed to derive a high-performing model for predicting mild and severe distress groups as per the feedback of breast cancer survivors. The data were randomly divided at the ratio of 7:3 into a training dataset for building the predictive model through learning and testing dataset for validating the built predictive model. The training dataset was divided into five subsets and k-fold cross-validation was performed to mitigate overfitting and improve the robustness. Moreover, a grid search was conducted to identify the optimal hyperparameters for each model. This task was performed using the train_test_split and GridSearchCV functions in scikit-learn, which is a representative machine learning package.

#### Model testing

Model testing is the process of evaluating the performance of a machine learning model constructed through training^19^. In this study, the accuracy, precision, recall, F1 score, and AUC were used as the performance metrics, where higher values indicate better predictive power of the model. Accuracy is the ratio of data that were correctly predicted in the mild and severe distress groups. Precision indicates the ratio of subjects who were actually experiencing severe distress among those who were predicted to be experiencing severe distress. Recall is the ratio of subjects predicted to be under severe distress among the subjects who were actually experiencing severe distress. The F1 score is the harmonic mean of the precision and recall and represents a high value when precision and recall are similar. Finally, the AUC score is an area, which is represented as a percentage. It indicates the effectiveness and generalization of the classification performance of the model^19^. This task was performed using the accuracy_score, precision_score, recall_score, f1_score, and roc_auc_score functions in scikit-learn.

#### Feature importance

Feature importance is a metric that indicates the impact of each feature on predictions during the training of the machine learning model^19^. In this study, the top-10 variables with high relative importance were extracted and visualized based on the coefficient using the feature_importances_ and coef_ functions in scikit-learn.

### Ethical considerations

This study was performed in accordance with the principles of the Helsinki declaration, and the procedures were followed in accordance with institutional guidelines. The study was approved by the institutional review board of the Ajou University (AJOUIRB-DB-2022–305), and all patients gave written informed consent.

## Results

### Demographic and treatment-related characteristics of participants

The average age of the participants was 53.3 years (± 9.0), with the highest proportion of participants in the 50 s age group, comprising 278 participants (43.4%). In terms of the disease stage at diagnosis, 274 participants (42.8%) were in stage 1, 234 participants (36.5%) were in stage 2, and 77 participants (12.0%) were in stage 3. The average duration since diagnosis was 33.9 months (± 18.4). All participants had undergone breast-cancer-related surgery, with 392 participants (61.2%) receiving chemotherapy, 603 participants (94.1%) receiving radiation therapy, 462 participants (72.1%) receiving hormone therapy, and 49 participants (7.6%) receiving targeted therapy (Supplementary Table 1).

### Characteristics according to the distress experienced by participants and distress problem list

The average distress score of the participants was 4.35 (± 2.38), with 271 participants (42.3%) recording mild distress scores of < 4 and 370 participants (57.7%) recording severe distress scores of ≥ 4 (Supplementary Table 1).

### Relationships between distress groups according to the demographic and treatment-related characteristics of the participants

When considering the relationships between distress groups based on the demographic and treatment-related characteristics of the participants (Table 1), no statistically significant differences were observed between the two groups in any of the characteristics.

**Table 1:** Demographic and treatment-related factors of patients in mild and moderate-severe distress groups.

Variable	Categories	Mild distress group (n = 271)	Moderate-severe distress group (n = 370)	t or χ^2^ (p)
		M ± SD or n (%)	M ± SD or n (%)	
Age (yr)	< 40	9 (26.5)	25 (73.5)	5.83 (.120)
	40–49	69 (38.8)	109 (61.2)	
	50–59	127 (45.7)	151 (54.3)	
	≥ 60	66 (43.7)	85 (56.3)	
Cancer stage	0	18 (32.1)	38 (67.9)	5.02 (.170)
	1	110 (40.1)	164 (59.9)	
	2	110 (47.0)	124 (53.0)	
	3	33 (42.9)	44 (57.1)	
Period after cancer diagnosis (m)		33.30 ± 13.40	34.30 ± 21.30	− 0.68 (.495)
Body mass index		23.70 ± 3.55	23.50 ± 4.03	0.58 (.564)
Chemotherapy	Yes	176 (44.9)	216 (55.1)	2.84 (.092)
	No	95 (38.2)	154 (61.8)	
Radiation therapy	Yes	260 (43.1)	343 (56.9)	2.94 (.086)
	No	11 (28.9)	27 (71.1)	
Hormone therapy	Yes	189 (40.9)	273 (59.1)	1.27 (.260)
	No	82 (45.8)	97 (54.2)	
Targeted therapy	Yes	25 (51.0)	24 (49.0)	1.66 (.197)
	No	246 (41.6)	346 (58.4)	

### Relationships between distress groups according to the distress problem list of the participants

The relationships between distress groups according to the list of distress problems selected by the participants are listed in Table 2. In terms of real-life problems, among the participants who responded that they had practical problems relating to child care (χ2 = 16.50, *p* < 0.001), housing (χ2 = 16.30, *p* < 0.001), insurance/financial aspects (χ2 = 12.10, *p* < 0.001), and work/school (χ2 = 7.47, *p* = 0.006), a higher proportion experienced severe distress rather than mild distress. In terms of family problems, among participants who responded that they had problems relating to dealing with children (χ2 = 12.80, *p* < 0.001), dealing with a partner (χ2 = 30.90, *p* < 0.001), and family health issues (χ2 = 5.26, *p* = 0.022), a higher proportion were classified in the severe distress group. In terms of emotional problems, a higher proportion of participants in the severe distress group reported problems with depression (χ2 = 38.60, *p* < 0.001), fears (χ2 = 25.40, *p* < 0.001), nervousness (χ2 = 20.80, *p* < 0.001), sadness (χ2 = 17.30, *p* < 0.001), worry (χ2 = 22.50, *p* < 0.001), and loss of interest in usual activities (χ2 = 15.90, *p* < 0.001). In terms of physical problems, a higher proportion of participants in the severe distress group reported problems with appearance (χ2 = 16.20, *p* < 0.001), eating (χ2 = 6.10, *p* = 0.013), fatigue (χ2 = 15.80, *p* < 0.001), getting around (χ2 = 5.52, *p* = 0.019), memory/concentration (χ2 = 17.10, *p* < 0.001), mouth sores (χ2 = 4.34, *p* = 0.037), dry/congested nose (χ2 = 3.90, *p* = 0.048), pain (χ2 = 8.20, *p* = 0.004), sleep (χ2 = 16.90, *p* < 0.001), tingling in hands/feet (χ2 = 4.47, *p* = 0.035), and spiritual/religious concerns (χ2 = 5.05, *p* = 0.025).

**Table 2:** Problem lists in mild and moderate-severe distress groups.

Domain	Variables		Mild distress group (n = 271)	Moderate-severe distress group (n = 370)	χ^2^	*p*
Practical problems	Child care	Yes	36 (26.9)	98 (73.1)	16.50	< .001
		No	235 (46.4)	272 (53.6)		
	Housing	Yes	7 (14.6)	41 (85.4)	16.30	< .001
		No	264 (44.5)	329 (55.5)		
	Insurance/financial	Yes	13 (21.3)	48 (78.7)	12.10	< .001
		No	258 (44.5)	322 (55.5)		
	Transportation	Yes	15 (60.0)	10 (40.0)	3.35	.067
		No	256 (41.6)	360 (58.4)		
	Work/school	Yes	20 (27.4)	53 (72.6)	7.47	.006
		No	251 (44.2)	317 (55.8)		
	Treatment decisions	Yes	15 (33.3)	30 (66.7)	1.59	.208
		No	256 (43.0)	340 (57.0)		
Family problems	Dealing with children	Yes	18 (23.4)	59 (76.6)	12.80	< .001
		No	253 (44.9)	311 (55.1)		
	Dealing with partner	Yes	19 (17.9)	87 (82.1)	30.90	< .001
		No	252 (47.1)	283 (52.9)		
	Ability to have children	Yes	0 (0.0)	5 (100.0)		.077*
		No	271 (42.6)	365 (57.4)		
	Family health issues	Yes	38 (32.8)	78 (67.2)	5.26	.022
		No	233 (44.4)	292 (55.6)		
Emotional problems	Depression	Yes	39 (22.4)	135 (77.6)	38.60	< .001
		No	232 (49.7)	235 (50.3)		
	Fears	Yes	47 (26.4)	131 (73.6)	25.40	< .001
		No	224 (48.4)	239 (51.6)		
	Nervousness	Yes	25 (22.7)	85 (77.3)	20.80	< .001
		No	246 (46.3)	285 (53.7)		
	Sadness	Yes	20 (22.2)	70 (77.8)	17.30	< .001
		No	251 (45.6)	300 (54.4)		
	Worry	Yes	96 (32.3)	201 (67.7)	22.50	< .001
		No	175 (50.9)	169 (49.1)		
	Loss of interest in usual activities	Yes	19 (22.4)	66 (77.6)	15.90	< .001
		No	252 (45.3)	304 (54.7)		
Spiritual/religious concerns	Spiritual/religious concerns	Yes	1 (9.1)	10 (90.9)		.029*
		No	270 (42.9)	360 (57.1)		
Physical problems	Appearance	Yes	38 (27.3)	101 (72.7)	16.20	< .001
		No	233 (46.4)	269 (53.6)		
	Bathing/dressing	Yes	14 (38.9)	22 (61.1)	0.18	.672
		No	257 (42.5)	348 (57.5)		
	Breathing	Yes	8 (25.8)	23 (74.2)	3.62	.057
		No	263 (43.1)	347 (56.9)		
	Changes in urination	Yes	7 (29.2)	17 (70.8)	1.76	.185
		No	264 (42.8)	353 (57.2)		
	Constipation	Yes	20 (36.4)	35 (63.6)	0.86	.353
		No	251 (42.8)	335 (57.2)		
	Diarrhea	Yes	11 (52.4)	10 (47.6)	0.91	.341
		No	260 (41.9)	360 (58.1)		
	Eating	Yes	36 (31.9)	77 (68.1)	6.10	.013
		No	235 (44.5)	293 (55.5)		
	Fatigue	Yes	128 (35.5)	233 (64.5)	15.80	< .001
		No	143 (51.1)	137 (48.9)		
	Feeling swollen	Yes	47 (36.2)	83 (63.8)	2.51	.113
		No	224 (43.8)	287 (56.2)		
	Fevers	Yes	33 (40.7)	48 (59.3)	0.09)	.764
		No	238 (42.5)	322 (57.5)		
	Getting around	Yes	23 (29.9)	54 (70.1)	5.52	.019
		No	248 (44.0)	316 (56.0)		
	Indigestion	Yes	47 (35.6)	85 (64.4)	3.03	.082
		No	224 (44.0)	285 (56.0)		
	Memory/concentration	Yes	46 (28.4)	116 (71.6)	17.10	< .001
		No	225 (47.0)	254 (53.0)		
	Mouth sores	Yes	3 (17.6)	14 (82.4)	4.34	.046
		No	268 (42.9)	356 (57.1)		
	Nausea	Yes	27 (37.5)	45 (62.5)	0.76	.384
		No	244 (42.9)	325 (57.1)		
	Dry/congested nose	Yes	12 (27.9)	31 (72.1)	3.90	.048
		No	259 (43.3)	339 (56.7)		
	Pain	Yes	87 (35.2)	160 (64.8)	8.20	.004
		No	184 (46.7)	210 (53.3)		
	Sexual	Yes	7 (25.0)	21 (75.0)	3.58	.058
		No	264 (43.1)	349 (56.9)		
	Dry/itchy skin	Yes	35 (38.9)	55 (61.1)	0.49	.483
		No	236 (42.8)	315 (57.2)		
	Sleep	Yes	81 (32.3)	170 (67.7)	16.90	< .001
		No	190 (48.7)	200 (51.3)		
	Substance use	Yes	0 (0.0)	3 (100.0)		.267*
		No	271 (42.5)	367 (57.5)		
	Tingling in hands/feet	Yes	59 (35.3)	108 (64.7)	4.47	.035
		No	212 (44.7)	262 (55.3)		

*Fisher’s exact test.

### Comparison of distress-predicting performance for participants using machine learning models

**Figure 1:**
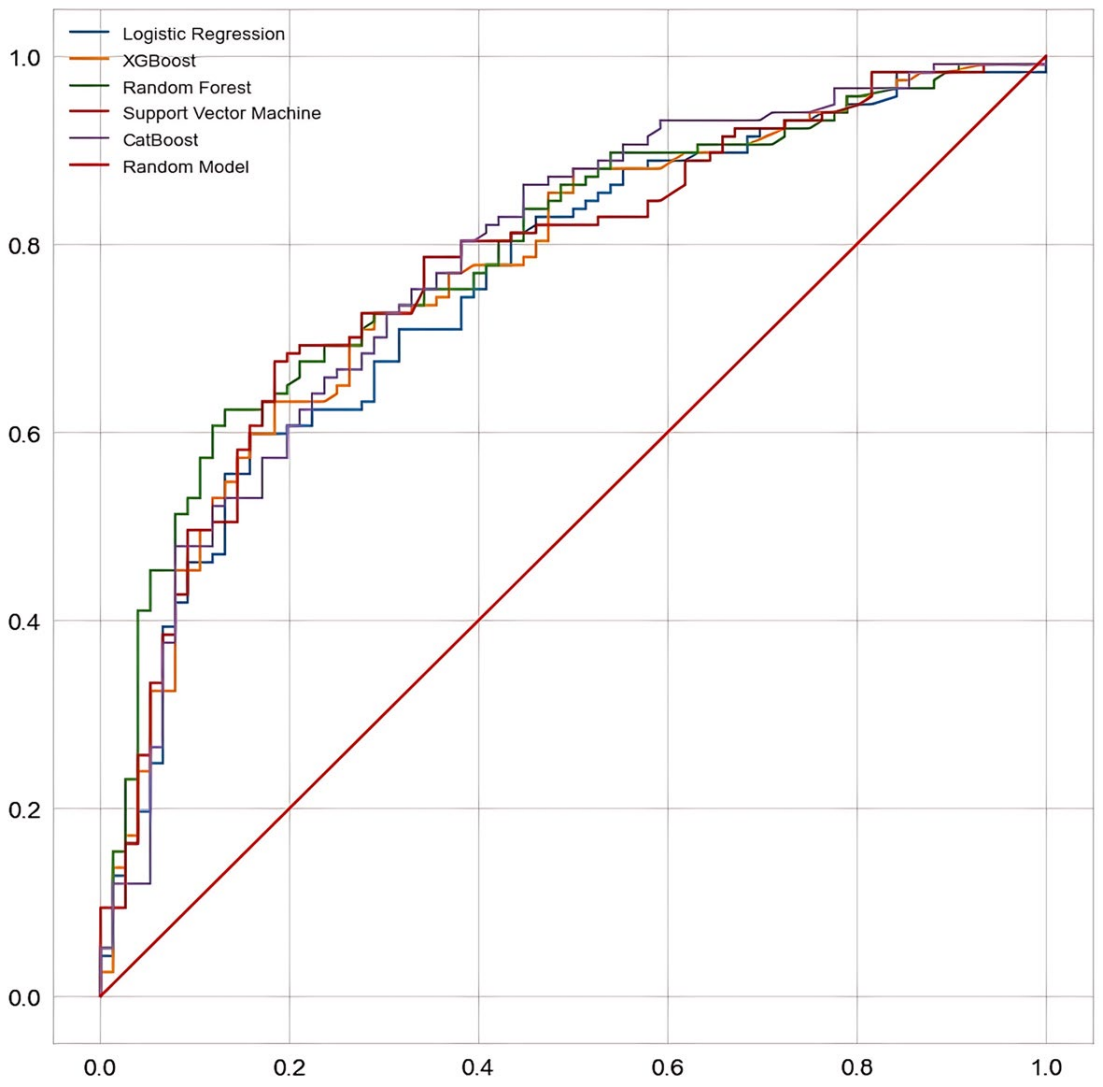
Comparative analysis of distress prediction performance in breast cancer survivors using machine learning models based on AUC scores.

The results of the various machine learning models and a comparison of their performances when determining the factors influencing the distress of participants are listed in Table 3. The accuracy scores of the Support Vector Machine, XGBoost, and CatBoost models were similar, with a value of 0.715. In terms of precision, the Support Vector Machine exhibited the highest value of 0.810, followed by XGBoost with 0.792 and CatBoost with 0.787. Moreover, Random Forest achieved the highest performance in terms of recall (0.821), followed by CatBoost (0.726) and XGBoost (0.718). Similarly, Random Forest exhibited the highest performance in terms of F1 score (0.771), followed by CatBoost (0.756) and XGBoost (0.753). The area under the receiver operating characteristic curve (AUC) score indicates that Support Vector Machine (0.721), XGBoost (0.714), and CatBoost (0.712) achieved the best performances in descending order (Fig. 1).

**Table 3:** Comparison of predictive performance for distress in breast cancer survivors using machine learning models.

	Accuracy	Precision	Recall	F1 score	AUC score
Logistic Regression	0.694	0.774	0.701	0.735	0.693
XGBoost	0.715	0.792	0.718	0.753	0.714
Random Forest	0.705	0.727	0.821	0.771	0.673
Support Vector Machine	0.715	0.810	0.692	0.747	0.721
CatBoost	0.715	0.787	0.726	0.756	0.712

### Importance of the top-10 variables derived from the Support Vector Machine, XBGoost, and CatBoost models for predicting the distress group of breast cancer survivors

**Figure 2:**
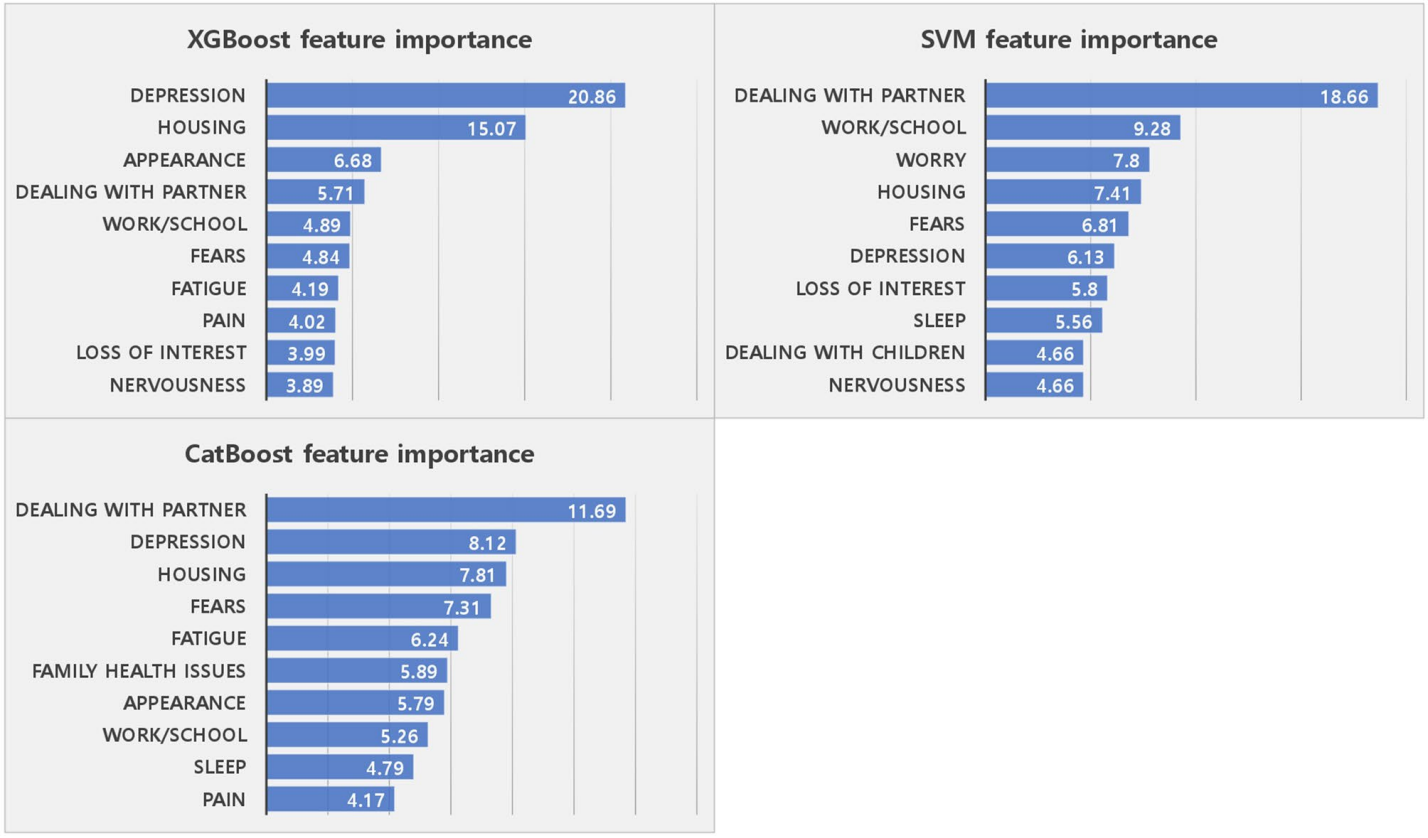
Top-10 features in order of importance as calculated using XGBoost, Support Vector Machine, and CatBoost.

The Support Vector Machine model showed the highest predictive performance based on the AUC score. The importance of the variables was ranked in the following order: dealing with a partner, work/school, worry, housing, fears, depression, loss of interest in normal activities, sleep, dealing with children, and nervousness. The XGBoost algorithm, which is known for its superior predictive performance, ranked the variables in descending order of importance as follows: depression, housing, appearance, dealing with a partner, work/school, fears, fatigue, pain, loss of interest in usual activities, nervousness. CatBoost, which was ranked third in terms of performance, rated the variables in descending order of importance as follows: dealing with a partner, depression, housing, fears, fatigue, family health difficulties, appearance, work/school, sleep, and pain (Fig. 2).

## Discussion

When the machine learning models were applied to identify the factors for predicting distress in breast cancer survivors, Support Vector Machine, XGBoost, and CatBoost demonstrated superior predictive performance in terms of the AUC score. The factors that were determined as significant predictors were depression among emotional problems, dealing with a partner, housing and work/school among practical problems, and fatigue among physical problems.

The results of this study showed that the distress level of breast cancer patients after completing primary treatment was an average of 4.35 points, and the proportion of participants who were classified under severe distress based on the score of 4 points, which is specified in the NCCN guidelines, was 57.7%. Distress in breast cancer patients occurs from the time of diagnosis until the completion of treatment, and it persists in approximately one-third to one-half of patients even after the completion of primary breast cancer treatment^8^. The time after the completion of treatment is crucial for the adaptation of cancer survivors. However, at this point, patients who have experienced distress may have a slow recovery process and persistent physical and psychological symptoms^20^. During this period, breast cancer patients who experience ongoing distress may not only experience delays in adaptation and recovery in their daily lives^21,22^, but also face difficulties in readjusting to societal requirements, such as returning to work, thereby resulting in a lower quality of life. However, note that many breast cancer patients do not receive any specific management beyond regular hospital visits after their treatment is completed. Therefore, the level of distress in breast cancer patients when primary treatment has been completed should be assessed, and concrete and practical measures to alleviate it should be determined.

The high-performance machine learning models Support Vector Machine, XGBoost, and CatBoost were applied to identify the predictive factors of distress in breast cancer survivors. The results showed that the accuracy, F1 score, and AUC score of these models were considerably high, exceeding 0.70. The Support Vector Machine, XGBoost, and CatBoost models have been reported to demonstrate superior predictive performance in multiple studies on the prediction of psychological symptoms such as distress^23–25^. Support Vector Machine is an algorithm that identifies the boundary with the largest margin by setting a hyperplane between the data, and it exhibits low overfitting and superior classification performance^26^. The XGBoost model is an ensemble model of decision trees that achieves fast learning and classification speeds using parallel processing. It also exhibits superior predictive performance in classification and regression^27^. Furthermore, the CatBoost model achieves high accuracy for categorical variables using ordered boosting^28^. This study demonstrates that, compared with the traditional binary classification method of logistic regression, machine learning models exhibit not only improved overall performance metrics but also offer a more intuitive understanding of the relationships between multiple variables and their feature importance. Particularly, the Support Vector Machine model demonstrated superior classification performance relative to ensemble models such as XGBoost and CatBoost. This is attributed to the parallel combination of single models in ensemble methods, which can lead to issues such as increased computational time and overfitting^19^. The Support Vector Machine, with its sequential learning process, mitigates these issues. Furthermore, it exhibits high generalization performance on new data and robustness to outliers^19^, making it highly beneficial for clinical settings where identifying and understanding a multitude of factors is crucial.

Regarding the importance of variables in predicting distress among breast cancer survivors, the results of the Support Vector Machine, XGBoost, and CatBoost models indicated that emotional problems such as depression, fears, worry, loss of interest in usual activities, and nervousness are significant predictive factors. Emotional symptoms such as depression, fear, and anxiety are reported to occur in breast cancer patients in a complex and clustered manner^29^. These symptoms are observed from the time of diagnosis and can last for more than 10 years after treatment has ended^30^^.^ These emotional issues have been identified as the most important variables that influence the quality of life and adaptation of breast cancer patients following primary treatment^22,31^. Considering that the association between these emotional symptoms and long-term survival rates in cancer patients has been established^32^, emotional symptoms must be monitored continuously and comprehensive approaches for mental health promotion, such as psychological support and counseling specifically designed for breast cancer survivors, must be implemented.

Furthermore, depression has a higher prevalence rate than other emotional symptoms^29^ and is considered the most influential factor in impairing the return of patients to routine life^32^. In particular breast cancer patients who have completed primary treatment experience a decrease in attention and support from family and friends compared to that during the treatment period, which leads to a more severe level of depression in a psychologically and socially vulnerable state. Such high levels of depression may hinder the return of breast cancer patients to normal life, affecting their adaptation and transition as survivors, as well as increasing the risk of recurrence and mortality^33,34^.Therefore, efforts are required to detect depression promptly and deal with it. effectively.

The Support Vector Machine, XGBoost, and CatBoost models identified dealing with a partner, housing, and work/school among practical problems as the most influential factors, demonstrating superior predictive performance. The results indicated that breast cancer survivors with problems dealing with partners experienced higher distress. Close interaction with caregivers is a crucial source of emotional support for breast cancer patients during surgery and treatment, as well as during the post-treatment period when they return to their daily lives and adapt to various changes. This interaction plays a significant role in managing the physical and psychological issues caused by post-treatment symptoms, and it enhances the overall well-being and adjustment of the patients^35^. Previous studies have indicated a positive impact on the psychological and social adaptation of breast cancer patients when they experience a high level of intimacy with their partners^36,37^. Thus, we can conclude that strengthening the intimacy with a partner is an important intervention factor in alleviating distress.

In terms of housing, unstable housing situations of breast cancer survivors may arise from financial risks associated with cancer diagnosis and treatment, particularly among low-income individuals^38^. The housing issue, which has a significant impact on family economics, lowers the living standards of households, increases the risk of contracting diseases, exacerbates distress, and impairs treatment compliance^39^.

Another important influencing factor that was identified in this study is the distress associated with returning to work, which functions as a social and financial safety net^40^. “Return to work” serves as an indicator of improved self-esteem in breast cancer survivors and signifies their social reintegration from being patients to becoming survivors^41^. Furthermore, the increase in income by returning to work is an important aspect of cancer recovery, providing economic stability and a sense of security for breast cancer survivors^42^. However, many breast cancer survivors experience difficulties in the process of returning to work owing to various physical, psychological, and social issues caused by treatment^43^. After returning to work, cancer survivors face several challenges such as a decrease in social status or rank, unwanted job replacements, issues with employers and colleagues, and diminished physical abilities. These difficulties make it challenging for them to continue working and cause distress^40,43^. Thus, practical support measures must be established to assist breast cancer survivors in successfully returning to work and achieving economic stability^42^.

Finally, the machine learning models identified physical symptoms such as fatigue, sleep, and pain as key predictors of distress among breast cancer survivors. Fatigue, sleep disorders, and pain are frequently reported as a symptom cluster in breast cancer survivors and can persist for more than five years^44^. Moreover, the symptom cluster in breast cancer survivors exhibits various patterns depending on the severity of the symptoms, affecting physical and social functioning, thereby leading to distress and reducing quality of life^45^. Consequently, for breast cancer survivors to return to their normal lives successfully following completion of primary treatment, intervention is required to monitor and manage the symptoms experienced by these survivors effectively.

This study is significant as it assessed the level of distress in breast cancer survivors and identified factors that influence the widespread occurrence of distress using machine learning techniques. However, the findings of this study must be interpreted with caution because of the following limitations. First, the research findings cannot be generalized because this study was conducted only on breast cancer survivors registered at the Cancer Survivor Integration Support Center located in the Gyeonggi region of South Korea. Therefore, future research calls for an expanded nationwide investigation of research participants to assess the distress of breast cancer survivors in detail. Second, this study exhibited limitations in identifying the changes in distress experienced during the transition from breast cancer patients to breast cancer survivors and determining the factors that influence these changes. Therefore, a longitudinal study should be conducted to identify the patterns of distress changes and related factors in breast cancer survivors.

## Conclusions

This study demonstrated that approximately 50% of breast cancer patients experience distress. Factors affecting the distress of breast cancer survivors, including emotional problems such as depression, practical problems such as dealing with a partner, housing, work/school, and physical problems such as fatigue, were identified using machine learning models. When applied to hospital information systems in the future, the developed and validated model for screening severe distress in breast cancer patients can provide evidence for identifying individual factors and recommend customized interventions for high-risk groups of breast cancer patients who are experiencing severe distress.

### Supplementary Information

Supplementary Information.

## Data Availability

The dataset is not publicly available owing to conditions of the ethics approval. Data on a cohort level may be made available by the corresponding author upon reasonable request.
