# Supplementary material for: Factors influencing psychological distress among breast cancer survivors using machine learning techniques
Source: Sci Rep. 2024 Jul 1;14:15052. doi: 10.1038/s41598-024-65132-y (PMC11219858; doi:10.1038/s41598-024-65132-y)
Supplement: Supplementary file 1 — Supplementary Information. [file 41598_2024_65132_MOESM1_ESM.docx]

| Variable | Categories | n(%) | M±SD |
| --- | --- | --- | --- |
| Age(y) | <40  40-49  50-59  ≥60 | 34(5.3)  178(27.7)  278(43.4)  151(23.6) | 53.3±9.0 |
| Stage | 0 | 56(8.7) |  |
|  | 1 | 274(42.8) |  |
|  | 2 | 234(36.5) |  |
|  | 3 | 77(12.0) |  |
| Period after cancer diagnosis (m) |  |  | 33.9±18.4 |
| BMI |  |  | 23.6±3.8 |
| Surgery | Yes | 641(100) |  |
|  | No | 0(0) |  |
| Chemotherapy | Yes | 392(61.2) |  |
|  | No | 249(38.8) |  |
| Radiation therapy | Yes | 603(94.1) |  |
|  | No | 38(5.9) |  |
| Hormone therapy | Yes | 462(72.1) |  |
|  | No | 179(27.9) |  |
| Targeted therapy | Yes | 49(7.6) |  |
|  | No | 592(92.4) |  |
| Distress | <4 | 271(42.3) | 4.35±2.38 |
|  | ≥4 | 370(57.7) |  |

Supplementary Table1. Demographic and Treatment-related factors of Breast Cancer Survivors
